# The Particle Shape of WC Governing the Fracture Mechanism of Particle Reinforced Iron Matrix Composites

**DOI:** 10.3390/ma11060984

**Published:** 2018-06-11

**Authors:** Zulai Li, Pengfei Wang, Quan Shan, Yehua Jiang, He Wei, Jun Tan

**Affiliations:** 1 School of Materials Science and Engineering, Kunming University of Science and Technology, Kunming 650093, China; lizulai@126.com (Z.L.); wangpengfei0530@126.com (P.W.); jiangyehua@kmust.edu.cn (Y.J.); 18213053069@163.com (H.W.); 2 College of Materials Science and Engineering, Chongqing University, Chongqing 400030, China

**Keywords:** tungsten carbide, microstructure, mechanical properties, fracture mechanism, second phases, particle reinforced composites

## Abstract

In this work, tungsten carbide particles (WC_p_, spherical and irregular particles)-reinforced iron matrix composites were manufactured utilizing a liquid sintering technique. The mechanical properties and the fracture mechanism of WC_p_/iron matrix composites were investigated theoretically and experimentally. The crack schematic diagram and fracture simulation diagram of WC_p_/iron matrix composites were summarized, indicating that the micro-crack was initiated both from the interface for spherical and irregular WC_p_/iron matrix composites. However, irregular WC_p_ had a tendency to form spherical WC_p_. The micro-cracks then expanded to a wide macro-crack at the interface, leading to a final failure of the composites. In comparison with the spherical WC_p_, the irregular WC_p_ were prone to break due to the stress concentration resulting in being prone to generating brittle cracking. The study on the fracture mechanisms of WC_p_/iron matrix composites might provide a theoretical guidance for the design and engineering application of particle reinforced composites.

## 1. Introduction

Recently, particle reinforced metal matrix composite coating (also named particle reinforced metal matrix surface composites, PRMMSC) has attracted extensive attentions because a metal surface without coating can easily to suffer abrasion causing the degradation or failure of materials [1]. It is necessary and important to improve the surface properties such as mechanical properties (like strength, toughness and wear-resistance) and chemical properties (corrosion-resistance and oxidation-resistance) for prolonging the service life or minimizing loss of production [2,3,4,5,6,7,8,9,10]. Recently, the WC_p_/iron matrix surface composites have been extensively used in slurry pump, slurry elbow pipe, liner plate, roll fitting and so forth. These composites can be fabricated by cast infiltration [2,11,12], powder metallurgy [3], laser cladding [5,6,13,14,15,16,17], and so on, to generate great metallurgical bonding between the surface composite layer and the substrate due to the perfect wettability between WC_p_ and molten ferrous alloy.

In recent years, a large number of researchers have carried out plenty of studies on the mechanical properties of metal matrix composites varying with the particle concentration, particle size, stress state, temperature and so on [2,4,11,18,19,20,21,22,23]. However, particle shape is also one of the most important geometric factors for the reinforcement and it can thus affect the overall performance of composites. It is generally believed that cracks in PRMMSC part manufacturing are crucial to the reliable material properties, especially for the reinforcement particles with different shapes. A finite element method was used to evaluate the effects of particle shape (spheres, regular octahedra, cubes or regular tetrahedra) on the mechanical properties of particle reinforced composites and found that particles with different shapes and equal sizes affected the yield stress at different extent [24]. Rasool et al. discussed the effects of particle shape (spherical and non-spherical particle) on the macroscopic and microscopic linear behaviors (linear elastic, thermoelastic and thermal conduction responses) of particle reinforced composites by numerical methods [25]. Trofimov et al. found that 15 convex polyhedral particle shapes could change the effective elastic properties of particle-reinforced composites predicted using micromechanical homogenization and direct finite element analysis approaches [26].

Therefore, different shapes of reinforced particles can affect the mechanical properties of composites, resulting in different fracture modes for the composites. However, there are various shapes in the actual products of WC_p_, and they are bound to affect the mechanical properties of composites regarding reinforcement. Thus, in this work, WC_p_/iron matrix composites were prepared utilizing a liquid sintering technique, and the effects of WC particle shapes (taking spherical particle and irregular particle as examples) on the microstructure, mechanical properties and fracture mechanism for particle reinforced iron matrix composites were investigated in details.

## 2. Materials and Methods

### 2.1. Preparation of Composites

**Figure 1:**
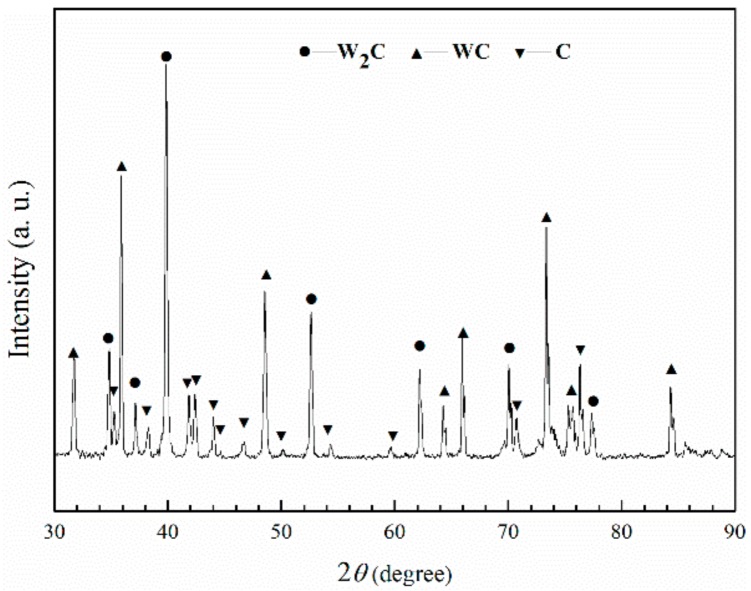
X-ray diffraction pattern of the as-received WC particles indicating that the particles were composed of W_2_C, WC and free carbon (C).

**Figure 2:**
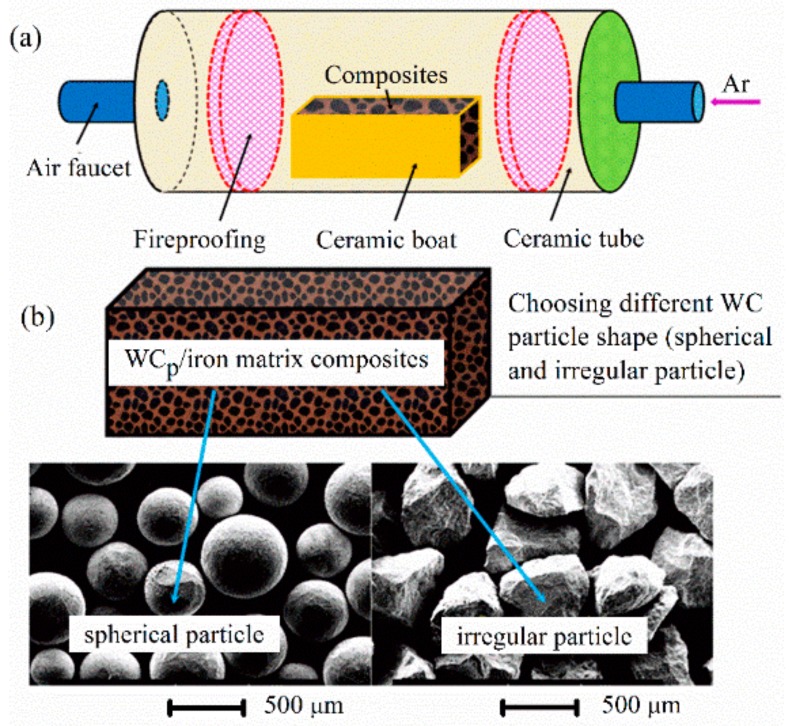
A schematic diagram of the preparation of WC_p_/iron matrix composites. (**a**) The heating schematic diagram of the tube furnace; (**b**) The morphology of spherical (left) and irregular (right) WC_p_.

The WC_p_/iron matrix composites were prepared utilizing a liquid sintering technique with the raw materials including WC_p_ and iron powders. The XRD pattern of the as-received WC powders is shown in Figure 1. It is clear that the as-received WC particles were composed of W_2_C, WC and free carbon (C). The schematic diagram of the WC_p_/iron matrix composites and the morphology of the WC_p_ are illustrated in Figure 2. WC_p_ and iron powders were firstly mixed by XQM-4L planetary ball mill (Nanjing Daran Technology Corporation, Nanjing, China), and it could make sure that WC_p_ would distribute in the iron powder uniformly. After that, the mixed powders were filled into a steel mold and then were pressed to form a green compact by manual hydraulic press with a pressure of 40 MPa for 60 min. The green compact was then placed into a corundum boat (100 mm × 56 mm × 35 mm). Later, it was placed into a tube furnace. The heating schematic diagram of the tube furnace was shown in Figure 2a. The process parameters of composites were described in Table 1. The heating rate of the vacuum tube furnace with a furnace pipe diameter of 80 mm (GSL-1600X, Kejing Company, Hefei, China) was in the range of 0–20 °C/min, operated at 220 V and 5.5 kW. Before being heated, the tube furnace was purged with high pure argon and then exhausted at least three times to protect the samples from pollution, and the vacuum valve was then closed when the pressure reached about 30 MPa. Finally, the heating temperature of the samples was elevated to 1500 °C, and kept for 60 min to make the interface react adequately. These samples were then naturally cooled in the furnace. Accordingly, the WC_p_/iron matrix composites with different shapes WC_p_ were prepared.

### 2.2. Characterization

The relative density of composites reinforced by spherical particles and irregular particle was 89.2 ± 1.0 and 88.6 ± 1.0 vol %, respectively. There were no obvious differences within the resolution limits of relative density measurement. The phase composition of these samples was characterized utilizing X-ray diffractometer (XRD, Empyrean, Panalytical Company, Almelo, The Netherlands) with a Cu-K*α* radiation operated at 40 kV and 30 mA. These samples were scanned in the 2*θ* range of 30–90°. Data were collected in a continuous mode with a scanning step of 0.02° and a time interval of 1 s/step. The microstructure of these samples was analyzed with scanning electron microscopy (SEM, VEGA 3 SBH, TESCAN, Brno, Czech Republic) combined with Energy Dispersive Spectrometer (EDS, GENESIS, EDAX, Mahwah, NJ, USA). Hardness of the samples was measured using a Rockwell hardness tester (FR-45, Laizhou Laihua Testing Instrument Factory, Laizhou, China) under a load of 150 kgf (1471 N) with a diamond cone indenter and duration of the test force 10 s. Each test was repeated at least 5 times, the value would be averaged. The compression tests were carried out by utilizing AG-IS 10 KN mechanical testing machine (Shimadzu Corporation, Kyoto, Japan). To ascertain reproducibility, each test result reported in this work was averaged from eight compression test under the same conditions. Finally, the fracture morphology of composites was observed using field emission scanning electron microscopy (FE-SEM, Nova Nano SEM 450, FEI Company, Hillsboro, OR, USA).

## 3. Results

### 3.1. Microstructure

The WC_p_ were mainly composed of WC and W_2_C phase identified by XRD, shown in Figure 1. Referencing the W-C phase diagram and previous theoretical calculations, the temperature of WC decomposition reaction was around 1250 °C [12].

2WC→W_2_C + C. (1)

The reaction (1) could promote to generate more W_2_C [19]. The W_2_C would react with iron to generate Fe_3_W_3_C. According to our previous first principles calculation, the cohesive energy *E*_coh_ of reaction between W_2_C and Fe was −0.01 eV/atom [12].

3Fe + 3/2W_2_C→Fe_3_W_3_C + l/2C. (2)

According to thermodynamic theory, reaction (2) could occur spontaneously when the cohesive energy is negative. These two reactions promoted each other and led to the interface reaction between WC_p_ and iron matrix around 1341 °C. Meanwhile, WC_p_ could decompose partially at a high speed in the heating process, more products of reaction (1) could be generated. The enrichment of W_2_C could provide more reactants for reaction (2) to finalize more Fe_3_W_3_C concentrated in the local area around WC_p_ [27].

**Figure 3:**
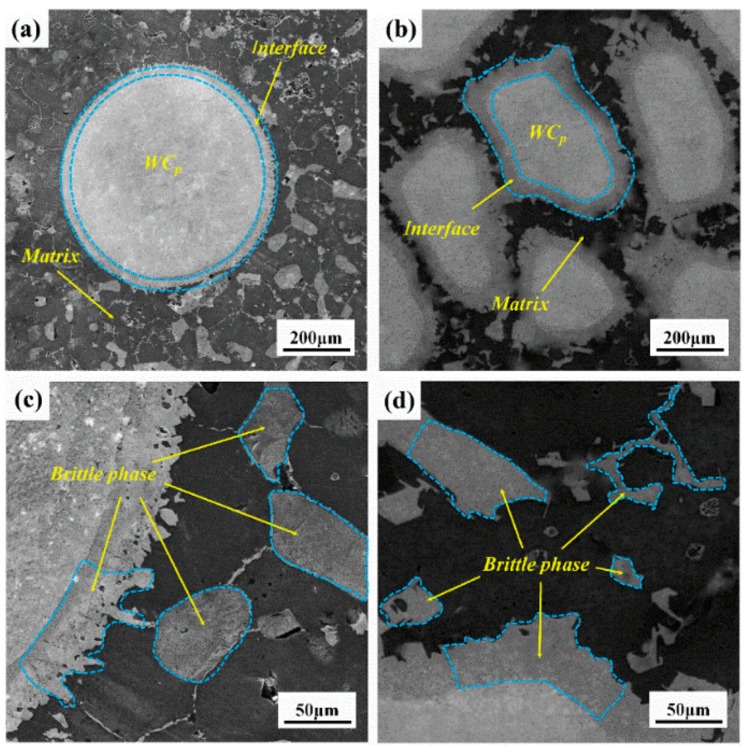
The metallographic photographs of composites with different particle shape: spherical particle (**a**,**c**), and irregular particle (**b**,**d**).

Spherical particles and irregular particles were evenly distributed in the matrix, and there was no aggregation. Irregular WC_p_ possessed more prominent edges and corners, while spherical WC_p_ presented regular sphere. The microstructure of prepared WC_p_/iron matrix composites with different particle shapes was shown in Figure 3. The spherical and irregular WC_p_ presented an integrated interface morphology state, and obvious interface reaction zones were generated in the surrounding, which demonstrated that particles occurred in the metallurgical reaction with iron matrix, shown in Figure 3a. A large number of brittle phase Fe_3_W_3_C was presented in the matrix with dispersed state. Comparing Figure 3a,b, the brittle phase Fe_3_W_3_C in spherical WC_p_/iron matrix composites was more homogeneous than that in irregular WC_p_/iron matrix. A typical magnification view is shown in Figure 3c,d, where plenty of intermittent massive structures appeared in irregular WC_p_ due to the stress concentration, which scattered into the iron matrix. Most W_2_C in WC_p_ would react with Fe_3_W_3_C in WC_p_/iron matrix composites. The metallurgical reaction (2) occurred between W_2_C and Fe, while the remaining WC particles distributed in the matrix presenting dark areas. In spherical WC_p_/iron matrix composites the bright white part (i.e., W_2_C) of WC_p_ was more, while the dark part was less (i.e., non-dissolved WC). As shown in Figure 3c,d, the brittle phase Fe_3_W_3_C presented a block structure in matrix. As shown in Figure 3b, the flat shape WC_p_ in irregular WC_p_/iron matrix composites tended to be round, and there was a trend turning into regular (spherical) WC_p_ because irregular WC_p_ had many bulges. These bulges would take precedence over some of the other flats or recessed parts, so the irregular WC_p_ had a trend of turning into regular WC_p_. The thickness of interface was very thin ranging from 5 to 60 μm. The thin interface was beneficial to transmitting the stress from matrix to WC_p_. How did this kind of reaction zone between interface affect mechanical properties?

### 3.2. Mechanical Properties

**Figure 4:**
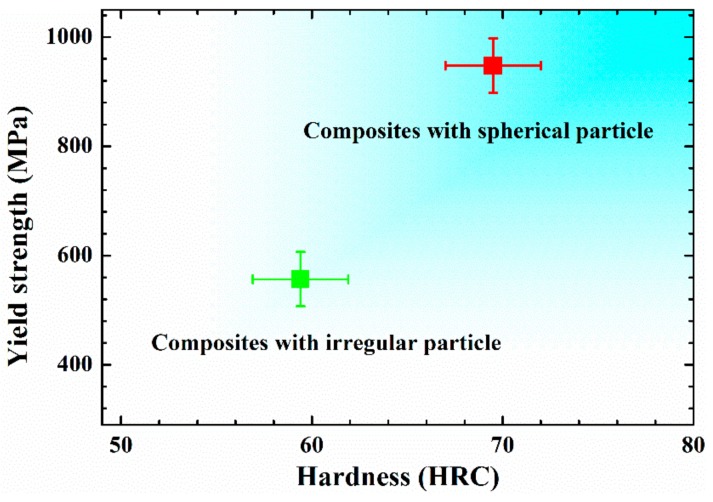
The mechanical properties of WC_p_/iron matrix composites with different particle shape. The error bars in this figure are the 1/2 intervals of the deviation of the minimum and maximum value.

The mechanical properties of WC_p_/iron matrix composites with different particle shape were tested at least eight times. As shown in Figure 4, the yield strength and hardness of spherical WC_p_/iron matrix composites were 947.8 ± 50 MPa and 69.5 ± 2.5 HRC, respectively. Under corresponding process parameters, the yield strength and the hardness of irregular WC_p_/iron matrix composites were 556.8 ± 50 MPa and 59.4 ± 2.5 HRC, respectively. 

Apparently, the spherical WC_p_/iron matrix composites had higher compression yield strength and hardness in comparison with the irregular WC_p_/iron matrix composites.

## 4. Discussion

**Figure 5:**
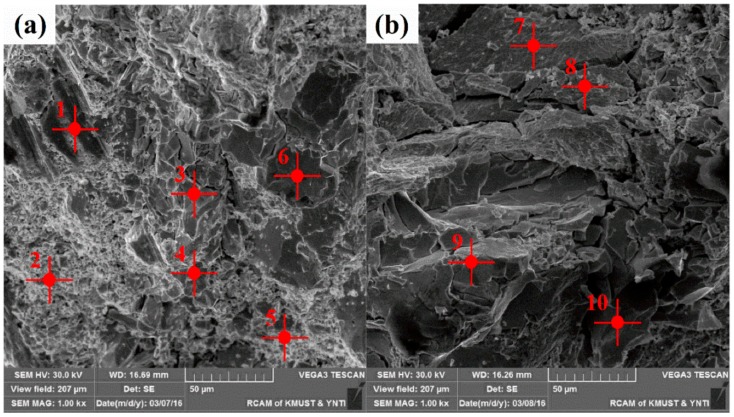
The compression fracture morphology of WC_p_/iron matrix composites with different particle shape: (**a**) spherical particle; (**b**) irregular particle.

In order to explore the initiation location of the micro-crack under compression test, SEM together with EDS analyses of different fracture location was carried out for spherical and irregular WC_p_/iron matrix composites. The initiation location of micro-crack in composites was determined by observing the phase composition of fracture location. According to the SEM photographs in Figure 5 and the EDS results summarized in Table 2, we could see that there were different element contents at points 1 and 2 in Figure 5a, with a higher Fe content and otherwise lower W and C content, so it could be speculated that these parts were a matrix of composites. At points 3, 4, 5 and 6, however, the atomic percentages of Fe and W were close to 1:1. Therefore, it could be speculated that the phase could be Fe_3_W_3_C, i.e., the location should be the interface of the composites. Micro-cracks could be found near points 3, 4, 5 and 6 in Figure 5a, so it could be inferred that the micro-cracks of spherical WC_p_/iron matrix composites initiated at the interface.

According to the Figure 5b and Table 2, the main compositions of irregular WC_p_/iron matrix composites were W and C, at points 7 and 8 in Figure 5b. It could be speculated that the phase was WC and W_2_C. Thus, the location was WC_p_ of composites. It meant that the brittle cracking occurred during compression tests. Because the convex portions of irregular WC_p_ were easier to produce stress concentration, the particles within composites were prone to cause brittle cracking [22]. The chemical composition of irregular WC_p_/iron matrix composites at points 9 and 10 could be recognized as Fe_3_W_3_C, because the atomic percentages of Fe and W were close to 1:1. This is to say that the location was the interface of composites. Micro-cracks, however, mainly initiated from points 7 and 8 in Figure 5b, so it could be speculated that the micro-cracks of irregular WC_p_/iron matrix composites initiated from the WC_p_ compound composed of WC and W_2_C.

Micro-cracks initiated near the interface of different shape WC_p_/iron matrix composites during compression tests. The micro-cracks extended into large cracks and resulted in the failure of composites. In the compression process, the irregular WC_p_ within composites tended to produce higher stress concentration in comparison with the spherical WC_p_, which were prone to cause brittle cracking.

**Figure 6:**
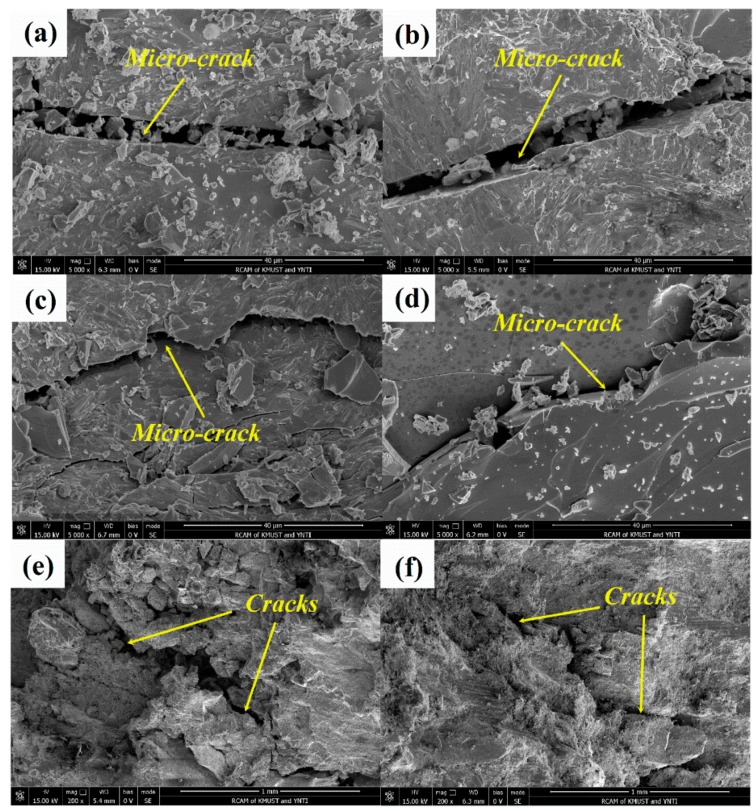
The fracture morphology of WC_p_/iron matrix composites with different particle shape: (**a**,**c**,**e**) spherical particle; (**b**,**d**,**f**) irregular particle.

The fracture morphology images of WC_p_/iron matrix composites with different particle shape are shown in Figure 6. From the fracture morphology images of spherical WC_p_/iron matrix composites in Figure 6a,c, it could be seen that there were not only obvious cleavage steps but also small dimples. However, the number of small dimples was limited, therefore, during compression tests, the fracture mode should be the quasi-cleavage fracture [4,18,28]. From the fracture morphology images of irregular WC_p_/iron matrix composites in Figure 6b,d, it could be seen that the matrix did not have plastic deformation before breaking, and the section was full of a cleavage step surface, so the fracture mode was a cleavage fracture (brittle fracture). This was because the content of interfacial phase Fe_3_W_3_C in the irregular WC_p_/iron matrix composites was higher than that in the spherical ones, and some Fe_3_W_3_C dissociated in the matrix existed as a brittle phase. It would increase the brittleness of composites, and make the spherical WC_p_/iron matrix composites present the transition mode by way of quasi-cleavage fracture to cleavage fracture [20,21,29]. The micro-cracks initiated and then expanded into a wider crack at the interface, resulting in the failure of the material. The compression strength of brittle fracture mode was lower than that of quasi-cleavage fracture mode for the composites. In this case, the yield strength of spherical WC_p_/iron matrix composites was 1.7 times of the irregular ones. The fracture surface of these samples after the compression test are shown in Figure 6e,f.

**Figure 7:**
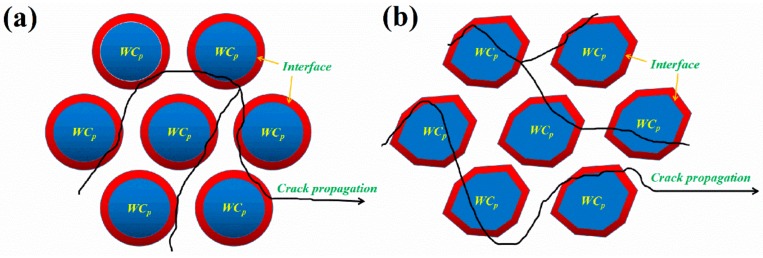
The crack propagations simulation diagram of WC_p_/iron matrix composites with different particle shape: (**a**) Spherical particle; (**b**) Irregular particle.

The crack propagation of WC_p_/iron matrix composites with different particle is schematically illustrated in Figure 7. It could be seen that the micro-cracks source of composites generated near the interface. Cracks initiated at the interface and expanded due to cohesive failure. Cracks could jump from one path to another when the fracture occurred. Several fracture paths might be produced when the cracks propagated through the matrix and encountered WC_p_. The cracks threaded entire irregular WC_p_ and resulted in the breakage of WC_p_ due to stress concentration. In fact, the irregular WC_p_ had many bulges, resulting in a bigger specific surface area. In the interfacial reaction zones, a more brittle Fe_3_W_3_C phase could be generated through diffusion. As discussed above, the brittle Fe_3_W_3_C phase was the root of crack initiation. This is to say that an irregular WC_p_ within the composites was prone to cause brittle crack. Therefore, the irregular WC_p_/iron matrix composites had lower yield strength and hardness. 

## 5. Conclusions

In summary, tungsten carbide particles (WC_p_) reinforced iron matrix composites with different shapes (spherical particles and irregular particles) were manufactured successfully by utilizing a liquid sintering technique. The effects of WC particle shape on the microstructure, mechanical properties and fracture mechanism for particle-reinforced iron matrix composites were investigated. The following conclusions could be drawn:

(1) In the interfacial reaction zone, WC particle and iron matrix could react into a brittle Fe_3_W_3_C phase.
(2) The spherical WC_p_/iron matrix composites had higher compression yield strength and hardness compared with the irregular ones.
(3) The micro-cracks source of composites were generated at the interface. The irregular WC_p_ within composites tended to produce a higher stress concentration compared with spherical WC_p_, which were prone to cause brittle fracture.
(4) Bigger specific surface area resulting from more bulges on irregular WC_p_ could lead to a more brittle Fe_3_W_3_C phase in the interfacial reaction zones. Therefore, the irregular WC_p_/iron matrix composites had lower yield strength and hardness.

## Figures and Tables

**Table 1:** The process parameters of WC_p_/iron matrix composites.

Particle Shape	WC_p_ Volume Fraction/%	Particles Size/μm	Holding Temperature/°C	Holding Time/min.
Spherical particle	40%	300–550	1500	60
Irregular particle	40%	300–550	1500	60

**Table 2:** The atomic percentage (at %) of WC_p_/iron matrix composites with different particle shape.

Point	Fe	W	C
1	85	5	10
2	87	4	9
3	43	40	17
4	43	39	18
5	43	39	18
6	43	40	17
7	2	63	35
8	3	62	35
9	43	40	17
10	43	40	17

## Nomenclature

PRMMSC	particle reinforced metal matrix surface composites
WC_p_	tungsten carbide particles
a. u.	arbitrary units
XRD	X-ray diffractometer
SEM	scanning electron microscopy
EDS	Energy Dispersive Spectrometer
HRC	Rockwell C hardness
FE-SEM	field emission scanning electron microscopy
